# Gray-Matter Expansion of Social Brain Networks in Individuals High in Public Self-Consciousness

**DOI:** 10.3390/brainsci11030374

**Published:** 2021-03-15

**Authors:** Tomoyo Morita, Minoru Asada, Eiichi Naito

**Affiliations:** 1 Institute for Open and Transdisciplinary Research Initiatives, Osaka University, 1-1 Yamadaoka, Suita, Osaka 565-0871, Japan; asada@otri.osaka-u.ac.jp; 2 Center for Information and Neural Networks (CiNet), National Institute of Information and Communications Technology (NICT), 2A6 1-4 Yamadaoka, Suita, Osaka 565-0871, Japan; eiichi.naito@nict.go.jp; 3 Graduate School of Frontier Biosciences, Osaka University, 1-1 Yamadaoka, Suita, Osaka 565-0871, Japan

**Keywords:** self-consciousness, emotion, social brain network, gray matter, voxel-based morphometry

## Abstract

Self-consciousness is a personality trait associated with an individual’s concern regarding observable (public) and unobservable (private) aspects of self. Prompted by previous functional magnetic resonance imaging (MRI) studies, we examined possible gray-matter expansions in emotion-related and default mode networks in individuals with higher public or private self-consciousness. One hundred healthy young adults answered the Japanese version of the Self-Consciousness Scale (SCS) questionnaire and underwent structural MRI. A voxel-based morphometry analysis revealed that individuals scoring higher on the public SCS showed expansions of gray matter in the emotion-related regions of the cingulate and insular cortices and in the default mode network of the precuneus and medial prefrontal cortex. In addition, these gray-matter expansions were particularly related to the trait of “concern about being evaluated by others”, which was one of the subfactors constituting public self-consciousness. Conversely, no relationship was observed between gray-matter volume in any brain regions and the private SCS scores. This is the first study showing that the personal trait of concern regarding public aspects of the self may cause long-term substantial structural changes in social brain networks.

## 1. Introduction

Self-consciousness is one of the personality traits that influences and determines one’s social attitudes and behaviors. To measure the degree to which individuals engage in self-consciousness, the Self-Consciousness Scale (SCS) questionnaire [1,2,3], originally developed by Fenigstein et al., has been widely used. This self-report questionnaire can measure at least two aspects of self-consciousness. One is public self-consciousness, which relates to caring about observable (overt) aspects of oneself, such as physical appearance and behaviors. The other is private self-consciousness, which relates to caring about internal (covert) aspects of oneself, such as inner thoughts and feelings.

It has been reported that individuals with a higher level of public self-consciousness tend to score high on SCS items such as “I am usually aware of my appearance” and “I am concerned about myself in the eyes of others”. Such people are not only concerned about their own appearance and behaviors but are also more sensitive and reactive to others’ gaze, reactions, attitudes, and evaluations of their physical appearance and behaviors [1,4]. They are also more likely to experience negative emotions (e.g., embarrassment and fear) in response to such evaluations by others [5,6]. Meanwhile, those with a higher level of private self-consciousness tend to score high on items such as “I often try to make sense of my own mind”. Such people tend to think and reflect a lot about themselves and are likely to make decisions and act according to their own inner thoughts and feelings [5].

Self-face images have been widely used to investigate neuronal correlates of public self-consciousness [7,8,9,10]. Previous functional magnetic resonance imaging (MRI) studies have reported that viewing self-face images (self-face recognition) consistently activates emotion-related brain networks such as the insular and cingulate cortices, in addition to the right inferior frontoparietal cortices (see meta-analysis in [11]). We previously reported that the emotion-related regions are particularly involved in the processing of embarrassment (one of the self-conscious emotions) that could often arise with self-face recognition [9,12]. In addition, we demonstrated that the emotion-related network augments its functional coupling with frontal midline structures, including the dorsal and ventral medial prefrontal cortex (MPFC), which is a key structure of the default mode network, when viewing self-face images, especially when the viewer knows that others also observe and evaluate the images at the same time [12].

As for private self-consciousness, thinking about internal aspects of the self (thoughts, traits, feelings) activates frontal and parietal midline structures, including the MPFC and precuneus of the default mode network [11,13,14,15]. Furthermore, recent neuroimaging studies showed that the temporal characteristics of spontaneous (resting-state) brain activity in the midline structures is correlated with the private SCS score [16,17]. Viewed collectively, we hypothesize that individuals with higher public and/or private self-consciousness, who likely recruit emotion-related and default mode networks frequently in their daily lives, show gray-matter (GM) expansions in these cortical regions.

To investigate this possibility, we collected structural MRI data from 100 young, healthy Japanese adults and performed a voxel-based morphometry (VBM) analysis. VBM allows the identification of brain structural changes that are associated with individual differences in personality traits, even in healthy volunteers [18,19,20]. In the present study, we used the Japanese version of the SCS [21], which was developed on the basis of the original questionnaire [1]. Sugawara (1984) selected specific questionnaire items with the idea to retain the two main factors most clearly related to the notions of public and private self-consciousness as originally defined by Fenigstein et al. (1975) [1,21]. We firstly searched for brain regions within the whole brain that showed GM expansion in relation to individual degrees of public and private self-consciousness. Herein, we expect gray-matter expansion in the emotion-related and/or default mode networks.

Secondly, to test the possibility that particular components of public and private self-consciousness are associated with these GM expansions, we performed additional analysis. Previous studies proposed the possibility of more than two (public and private) factors for self-consciousness because public and private self-consciousness can be further separated into several factors [22,23,24,25]. In the present study, we also expect several factors since the structure of self-consciousness is influenced by, among others, the period/era where participants answer the questionnaire, the culture to which participants belong, and the participants’ age [26,27,28]. Therefore, we conducted a factor analysis to explore the multiple possible factors of self-consciousness in young Japanese adults. We then sought to examine from which of the two original factors of self-consciousness (public and private) each of these additional factors originate. On the basis of these results, we examined the possibility of the existence of such subfactors that are particularly associated with the expansion in the emotion-related and/or default mode networks.

## 2. Materials and Methods

### 2.1. Participants

A total of 100 young, healthy Japanese adults (66 men and 34 women, mean age = 22.0 ± 1.6 years old) participated in this study. We recruited 50 right- and 50 left-handers from local universities. Their handedness was evaluated using the Edinburgh Handedness Inventory [29]. No participant had a history of neurological or psychiatric disorders. The protocol used for this study was approved by the ethics committee of the National Institute of Information and Communications Technology. We explained the details of the study to the participants before the start of the experiment. All participants provided written informed consent. We also obtained written informed consent from the legal guardians of the two participants aged under 20. The experiment was carried out following the principles and guidelines of the Declaration of Helsinki (1975).

### 2.2. Self-Consciousness Questionnaire

All participants were asked to answer the Japanese version of the SCS questionnaire [21], which was developed by Sugawara (1984) on the basis of the original scale [1]. Sugawara’s version consists of 11 and 10 items evaluating public and private self-consciousness, respectively. The public part of the SCS measures the dispositional tendency to be aware of the publicly displayed (observable) aspects of oneself, such as one’s physical appearance and behavior (e.g., *“I am concerned about my style of doing things”*). The private part of the SCS, on the other hand, measures the dispositional tendency to be aware of the covert and hidden (unobservable) aspects of oneself, such as one’s own thoughts and feelings (e.g., *“I am generally attentive to my inner feelings”*). Each participant rated each of the 21 items using a seven-point Likert scale from 1 “strongly disagree” to 7 “strongly agree”. We calculated the public SCS score of each participant by summing up the scores of all 11 items. The same was done for the private SCS score, for which we summed up the scores of all 10 items. These scores were used as covariates in the whole-brain multiple regression analysis of the structural MRI data. We reported Cronbach’s alpha [30] to show internal consistency for each of public and private SCSs. Data were collected from June to November 2016.

### 2.3. Factor Analysis

We conducted an exploratory factor analysis using the principal factor method on all 21 items for the SCS. We applied a varimax rotation, because this can produce factors that are orthogonal to each other. Through this procedure, we could obtain factor scores that are independent to each other and, thus, we could use these scores as independent variables in the following multiple regression analysis. SPSS statistics 24 (IBM SPSS Inc., Chicago, IL, USA) was used for all analyses. We identified orthogonal factors whose eigenvalues were greater than 1 according to the Kaiser–Guttman rule [31,32]. We identified five factors and calculated the factor score for each factor in each participant using all 21 item scores and their respective factor loadings. In order to examine the relationships among the five factors and each of the two original (public and private) factors, we performed an across-participant correlation analysis between the factor score for each of the five factors and the public or private SCS score. By doing so, we could confirm for each of these five factors which of the two original (public and private) factors of self-consciousness they were a subfactor of.

### 2.4. MRI Acquisition

For each participant, a T1-weighted magnetization-prepared rapid gradient echo (MP-RAGE) image was acquired using a 3 T MRI machine (Trio Tim; Siemens Healthineers, Erlangen, Germany) and a 32-channel array coil. The imaging parameters were as follows: repetition time (TR) = 1900 ms; echo time (TE) = 2.48 ms; flip angle (FA) = 9 degrees; field-of-view (FOV) = 256 × 256 mm^2^; matrix size = 256 × 256 pixels; slice thickness = 1.0 mm; voxel size = 1 × 1 × 1 mm^3^; 208 contiguous transverse slices.

### 2.5. VBM Analysis

We performed a voxel-based morphometry (VBM) analysis. This was done to explore possible expansion in GM volume in individuals who score high on public and/or private SCS. We first visually inspected the anatomical images of all participants and confirmed the absence of observable structural abnormalities and motion artefacts. The data were then processed using Statistical Parametric Mapping (SPM12, Wellcome Centre for Human Neuroimaging, University College London, London, UK). All steps were carried out as recommended by Ashburner (2010) [33]. First, the anatomical image obtained from each participant was segmented into GM, white matter (WM), cerebrospinal fluid (CSF), and nonbrain parts. The default settings in SPM12 were used for all parameters.

Next, using diffeomorphic anatomical registration through exponentiated lie algebra (DARTEL), we generated GM and WM DARTEL templates on the basis of anatomical images obtained from all participants. We used DARTEL in the default settings implemented in SPM12. We then applied an affine transformation to the GM and WM DARTEL templates to align them with their tissue probability maps in Montreal Neurological Institute (MNI) standard space. A segmented GM image from each participant was then nonlinearly warped to the GM DARTEL template in MNI space (spatial normalization). The warped image was modulated by Jacobian determinants of the deformation field to preserve relative GM volume even after spatial normalization. The modulated image of each participant was then smoothed with an 8 mm full-width-at-half-maximum (FWHM) Gaussian kernel and resampled to a resolution of 1.5 × 1.5 × 1.5 mm^3^ voxel size.

In the group-level analysis, to identify brain regions in which GM volume was positively correlated with public and private SCS scores respectively, we performed multiple regression analysis in the whole brain using the individual GM images. We included individual public and private SCS scores as independent variables, as well as sex, age, handedness score, and intracerebral volume (sum of GM, WM, and CSF volumes) as nuisance covariates (i.e., effects of no interest), since these factors may have a significant effect on the results of a VBM analysis [19]. To exclusively select truly positive GM voxels by eliminating possible noise outside the brain and to increase statistical power by reducing search volumes, we generated a mask on the basis of our present data using the SPM Masking Toolbox [34] (http://www0.cs.ucl.ac.uk/staff/g.ridgway/masking/; accessed on 18 March 2019). Thus, voxels outside of this mask were excluded from the analysis. We reported active voxels using a statistical height threshold of *p* < 0.005 and a statistical extent threshold of *p* < 0.05, corrected for multiple comparisons with the family-wise error rate (FWE) across the entire brain. For anatomical identification, we referenced the anatomical automatic labeling (AAL) atlas [35] and the cytoarchitectonic probability maps derived from the MNI standard brain in the SPM Anatomy Toolbox v2.2b [36].

**Figure 1:**
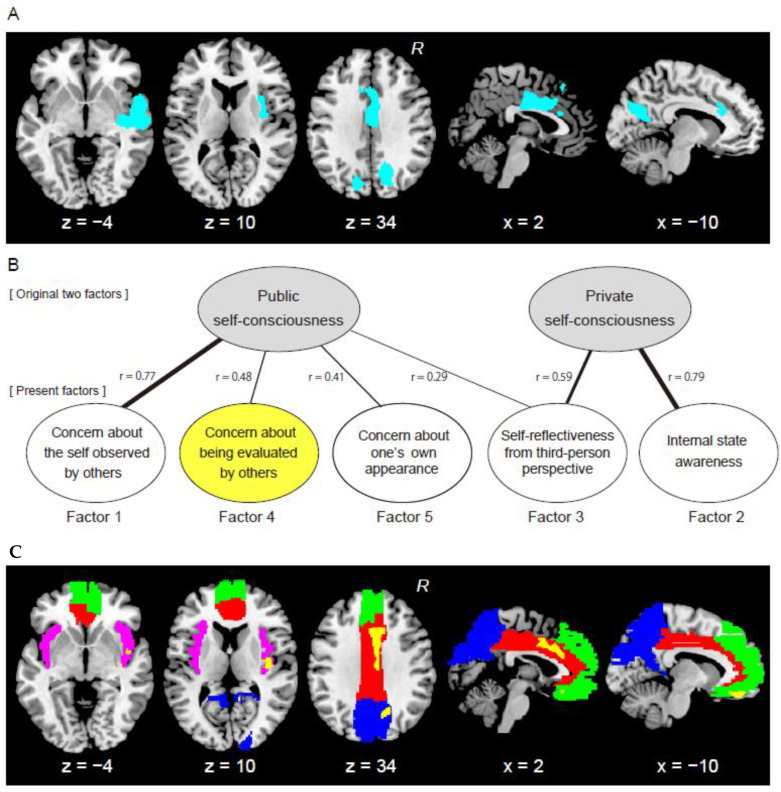
Summary of the factor analysis and voxel-based morphometry (VBM) analysis results. (**A**) Brain regions (cyan) in which gray-matter volume was positively correlated with public Self-Consciousness Scale (SCS) score. Images were superimposed onto the transvers and sagittal sections (z = −4, 10, and 34; x = 2 and −10) of a Montreal Neurological Institute (MNI) standard brain. We used a height threshold of *p* < 0.005 and an extent threshold of *p* < 0.05 family-wise error rate (FWE) corrected within the whole brain. (**B**) The five factors identified in the present study and their relationships with the original two (public and private) factors of self-consciousness. The thickness of the lines indicates correlation coefficients between the factor score for each of the five factors and the public or private SCS score (*r*, see details in the text). (**C**) Brain regions (yellow) in which gray-matter volume was positively correlated with the score of Factor 4, which was a subfactor of public self-consciousness, within four regions of interest (ROIs) (Insula ROI: magenta, anterior cingulate cortex/middle cingulate cortex (ACC/MCC) ROI: red, precuneus/cuneus ROI: blue, medial prefrontal cortex (MPFC) ROI: green). Images were superimposed onto the transverse and sagittal sections (z = −4, 10, and 34; x = 2 and −10) of an MNI standard brain. We used a height threshold of *p* < 0.005 (uncorrected) and an extent threshold of *p* < 0.05 corrected in each ROI (small volume correction). Abbreviations: R, right hemisphere.

Since the whole-brain analysis showed GM expansions in the emotion-related areas and the default mode networks in relation to the public SCS scores, we further examined the possibility of the existence of subfactors of the public self-consciousness that are particularly associated with these expansions. In this analysis, multiple regression analysis was performed with the same procedure as in the first multiple regression analysis except for independent variables. Within the five factors identified in the present factor analysis, we used individual factor scores of the three factors that were correlated with the public SCS score (correlation coefficient higher than 0.4) as independent variables. We selected four brain regions (Figure 1C) in the emotion-related network (insula, anterior cingulate cortex/middle cingulate cortex (ACC/MCC)) and the default mode networks (precuneus/cuneus and MPFC) as regions of interest (ROIs). Each anatomical ROI was created using the AAL atlas in Wake Forest University Pickatlas toolbox within SPM [37]. The insula ROI was defined as the bilateral “Insula” mask, and the ACC/MCC ROI was defined as the union of the bilateral “Cingulum_Ant” and “Cingulum_Mid” masks. Similarly, the precuneus/cuneus ROI was defined as the union of the bilateral “Precuneus” and “Cuneus” masks, and the MPFC ROI was defined as the union of the bilateral “Front_Sup_Medial”, “Frontal_Med_Orb”, and “Rectus” masks. Within these ROIs, we examined GM expansion in relation to the three factor scores. We reported active voxels (height threshold of *p* < 0.005) within each ROI with using small volume correction (SVC, *p* < 0.05 FWE [38]).

## 3. Results

### 3.1. Results from Whole-Brain Analysis

The average public SCS score across participants was 54.8 ± 9.3, whereas the average private SCS score was 50.4 ± 7.8. The Cronbach’s alphas for the public SCS and private SCS were 0.84 and 0.79, both of which were high enough to guarantee internal consistency and reliability of each SCS. In the whole-brain VBM analysis, we found brain regions in which GM volume was positively correlated with the public SCS scores in the anterior and middle cingulate cortices extending to the dorsal MPFC, in the right insular cortex extending into temporal cortex, and in the bilateral precuneus and cuneus (Figure 1A, Table 1). On the other hand, there were no brain regions in which GM volume was positively correlated with the private SCS scores. In addition, there were no regions in which GM volume was negatively correlated with the public and private SCS scores. The results indicated that brain regions in the emotion-related and defaults mode networks expanded in individuals with higher public self-consciousness.

### 3.2. Results from Factor Analysis

The exploratory factor analysis revealed five representative orthogonal factors. Each factor explained 14.1%, 14.1%, 11.1%, 6.9%, and 5.5% of the total variance, respectively. The matrix structure with the factor loadings and items is presented in Table 2. 

The items that had higher loadings on Factors 1, 4, and 5 were related to public self-consciousness, as defined by Sugawara (1984) [21]. The items with particularly high factor loadings for Factor 1 were items 6, 8, 9, 10, and 21 (more than the absolute value of 0.40). We named this “concern about the self observed by others”. The items with particularly high factor loadings for Factor 4 were items 18 and 21. Notably, some items (items 6, 8, and 10) showed higher loadings on Factor 1, while they showed lower loadings on Factor 4. When considering contents of these items, Factor 1 represents concern about the observable (overt) aspect of the self which could be observed by others, while Factor 4 represents concern about evaluation of oneself by others. Thus, Factor 4 is qualitatively different from Factor 1. Therefore, we named Factor 4 “concern about being evaluated by others”. Lastly, the items with particularly high factor loadings for Factor 5 were items 6 and 16. We named this “concern about one’s own appearance”.

When we examined correlations between the score of each of the three factors and the public SCS score, we found significantly positive correlations (*r* = 0.77, *r* = 0.48, and *r* = 0.41, for Factors 1, 4, and 5 respectively; *p* < 0.001 for all). However, when we examined correlations between the score of each of the three factors and the private SCS score, we found no significant correlation (*r* = 0.06, *r* = 0.05, and *r* = 0.10, respectively). This indicates that Factors 1, 4, and 5 can be considered as subfactors of public self-consciousness (Figure 1B).

The six items (1, 4, 5, 7, 15, and 17) with higher loadings on Factor 2 were all related to private self-consciousness as reported by Sugawara (1984) [21]. This factor seems to correspond to the private SCS subfactor “internal state awareness” previously reported by Mittal and Balasubramanian (1987) [23]. We, therefore, used the same name. We found a significantly positive correlation (*r* = 0.79, *p* < 0.001) between the score of Factor 2 and the private SCS score, but no significant correlation (*r* = 0.14) between this score and the public SCS score. This indicates that Factor 2 was related to private self-consciousness (Figure 1B).

Lastly, the items with particularly high factor loadings for Factor 3 were items 11, 12, and 14 that were related to private self-consciousness, and items 13 and 19 that were related to public self-consciousness. We named this factor “self-reflectiveness from a third-person perspective”. We found a significantly positive correlation (*r* = 0.59; *p* < 0.001) between the score of this factor and the private SCS score, as well as a weaker but still significant correlation (*r* = 0.29; *p* < 0.01) between this score and the public SCS score. Factor 3, thus, represents a mixed factor (Figure 1B), which was not reported in the original Japanese study [21].

### 3.3. Results from ROI Analysis

In the ROI analysis, we examined the possibility of existence of subfactors of public self-consciousness that were particularly associated with the GM expansions identified in the whole-brain analysis. We found that, among the three subfactors of public self-consciousness, the individual score of Factor 4 *“concern about being evaluated by others”* was correlated with the GM volume in the emotion-related and default mode network regions (Figure 1C and Table 3). A significant cluster of voxels showing a positive correlation with the score was identified in the ACC/MCC and precuneus/cuneus ROIs (SVC, *p* < 0.05). Clusters of voxels showing significant trend were also observed in the insula ROI (SVC, *p* = 0.07) and in the MPFC ROI (SVC, *p* = 0.08). In contrast, as for the other two subfactors (1 and 5) of public self-consciousness, we found no such significant GM expansion in any ROIs. Thus, the results indicated that the subfactor of public self-consciousness, i.e., the personal trait of concern about being evaluated by others, is particularly associated with the GM expansions in the emotion-related and default mode network regions.

## 4. Discussion

This study demonstrated that individuals scoring higher on the public SCS showed GM expansions in emotion-related regions of the cingulate and insular cortices, as well as in the default mode network involving the precuneus and MPFC. In addition, these GM expansions were associated with the personal trait of *“concern about being evaluated by others”*, which was identified as a subfactor of public self-consciousness. This indicates that the personal trait of concerns about public aspects of the self has a strong enough influence to generate long-term structural changes in social brain networks [39,40]. 

### 4.1. Gray-Matter Expansion

The ACC and the insular cortex are part of the emotion-related network. These regions are involved in the processing of a wide range of emotions, including not only basic but also more complex emotions elicited in a variety of social situations [41,42,43,44,45,46]. For example, the ACC and insular cortex become active when a person observes their own face [9,11,12,47,48], and their activity is further augmented when they experience negative emotion (embarrassment), such as in a situation where their face is simultaneously observed and evaluated by others [12].

The precuneus and the ventral and dorsal MPFC are also part of the default mode network [49] and are activated when participants think about the mental states of others [50]. Similarly, the MPFC becomes active when we think about how others evaluate us, such as when one’s appearance and behavior are evaluated by others [51,52,53]. In addition, the MPFC shows hyperactivity in individuals with social anxiety disorder who tend to ruminate upon negative evaluations received from others [54], when they view their own face images and feel strong negative emotions [6], as well as when they receive negative comments about themselves from others [55].

Importantly, ACC activity enhances its functional coupling with MPFC activity when a person experiences negative emotion in a social situation where their face was simultaneously evaluated by others [12]. Thus, the emotion-related and default mode network regions may become active simultaneously when a person experiences negative emotion when their appearance or behavior is evaluated by others. Moreover, the precuneus has been shown to be a hub region connecting the default mode network to emotion-related regions such as the ACC [56,57]. Hence, those who are highly concerned about public aspects of the self are likely to recruit emotion-related and default mode networks involved in social cognition (i.e., social brain [39,40]) frequently in their daily lives, and such a personal trait may expand GM in these cortical regions.

In contrast to the public SCS, the private SCS was not associated with GM volume in any brain region; we would have expected the private self-consciousness to expand the default mode network. The series of findings suggest that caring about observable (overt) aspects of the self has a stronger impact on neuronal processes in one’s brain than thinking about internal (covert) aspects of the self, presumably explained by the fact that humans are highly social beings.

### 4.2. Subfactors of Public Self-Consciousness

In the present study, we identified five factors of self-consciousness in young Japanese adults using the Japanese version of the SCS questionnaire [21]; Sugawara reported only two main independent factors (public and private). The identification of more than two self-consciousness factors when using the SCS questionnaire is in line with previous reports [22,23,24,25].

As for the three subfactors of public self-consciousness (Factors 1, 4, and 5), Factor 1, “concern about the self observed by others”, seems to be most similar to the original public factor, as this score was highly correlated with the public SCS score (*r* = 0.77). In addition, it is conceivable that Factor 5, “concern about one’s own appearance”, is similar to the factor “appearance consciousness” that was previously reported as a subfactor of public self-consciousness [23,24]. In contrast, Factor 4, “concern about being evaluated by others”, seems to be a novel factor. We assume that the development of a society that is highly and constantly networking, which increases one’s chance of being evaluated by others, might have contributed to the derivation of this new factor from the originally reported notion of public self-consciousness. The number of items showing high factor loadings might have been relatively small for this factor. However, this may not be a serious problem in the present VBM analysis because the analysis was conducted using not the scores calculated from the small number of selected items, but the factor scores calculated from the scores of all 21 items. In addition, given that only the score for this factor (Factor 4) was associated with the GM expansions in the emotion-related and default mode network regions that expanded in relation to the public SCS score, this must be an important subfactor composing public self-consciousness.

### 4.3. Factor 3

Factor 3 was determined to be mixed of both public and private, which is novel and has never been reported before. This seems to be a meaningful factor, since Cronbach’s alpha coefficient calculated from the scores of the five items (11, 12, 13, 14, and 19) with higher loadings to this factor showed a relatively high value (α = 0.75). Factor structures can vary depending, for example, on the time period/era when participants answer the questionnaire, on the culture to which participants belong, and on the participants’ age. Since the participants in Sugawara’s original study were in the same age group as in the current study, the major difference could be the era.

Previous studies reported two subfactors for private self-consciousness, i.e., *“internal-state awareness”* and *“self-reflectiveness”* [23,24]. We assume that Factor 3, *“self-reflectiveness from a third-person perspective”*, is derived from the latter by incorporating components of public self-consciousness. Self-reflection is originally a personal cognitive process within each individual, but it seems that it is currently becoming a social cognitive process that requires a third-person perspective. We conjecture that such a change could be related to the rapid development of social networking systems, which not only allows individuals to connect easily but also allows everyone to be constantly watched, observed, supervised, and evaluated by anonymous and familiar others.

## 5. Conclusions

We demonstrated that public self-consciousness, especially the personal trait of concern about being evaluated by others, was associated with the GM expansions in emotion-related and default mode network regions. To the best of our knowledge, this is the first study to show that personal trait affects neuronal processes in social brain networks in such a way that they lead to structural changes. This finding likely reflects the current society where individuals are frequently evaluated by anonymous and familiar others.

## Figures and Tables

**Table 1:** Brain regions with gray-matter expansions in individuals who scored higher on public SCS.

Clusters	Number of Voxels	Cluster-Level *p* (Corrected)	MNI Coordinates			T-Value	Anatomical Identification (Cytoarchitectonic Map)
			x	y	z		
Cingulate cluster	2172	0.03	3	−2	33	3.94	Rt MCC
			−12	24	32	3.27	Lt MCC
			0	27	50	3.09	Superior medial gyrus
			−6	27	24	3.02	Lt ACC
			2	26	23	2.98	Rt ACC
Rt temporal cluster	4206	<0.001	56	−6	−3	4.77	Superior temporal gyrus (Area TE 1.2)
			50	−17	0	4.34	Superior temporal gyrus
			56	8	−8	4.13	Temporal pole (Area TE 3)
			42	−20	−6	3.90	Insula (Area Id1)
			57	−20	−11	3.67	Middle temporal gyrus
			33	−3	14	3.47	Insula
			35	−14	−3	3.45	Insula (Area Ig2)
			53	8	−30	2.92	Temporal pole
Rt precuneus cluster	2359	0.02	17	−75	27	4.27	Cuneus
			11	−53	23	4.19	Precuneus
			24	−69	23	4.02	Superior occipital gyrus
Lt precuneus cluster	1859	0.06	−15	−57	26	4.40	Cuneus
			−17	−68	30	4.07	Superior occipital gyrus
			−14	−53	18	3.60	Precuneus

Abbreviations: Rt, right; Lt, left; MCC, middle cingulate cortex; ACC, anterior cingulate cortex.

**Table 2:** Questionnaire items of the Self-Consciousness Scale (SCS), identified factors, and their loadings.

	Items	Factor 1	Factor 2	Factor 3	Factor 4	Factor 5
**Public SCS items**						
i9	I am concerned about what other people think of me	**0.856**	0.029	0.141	0.257	0.024
i8	I am interested in rumors about myself	**0.754**	0.090	0.102	0.136	0.160
i10	I am concerned about myself in the eyes of others	**0.712**	0.247	0.128	−0.047	0.082
i6	Whenever I do something in public, I am concerned about my gestures and appearance	**0.540**	0.058	0.139	0.025	**0.502**
i2	I do not care about public opinion	−0.392	0.099	0.044	−0.352	−0.291
i19	I am concerned about how others perceive my statements	0.367	0.051	**0.586**	0.368	−0.126
i13	I am concerned about how to behave when I meet people	0.313	0.051	**0.586**	0.081	0.139
i18	I take care not to make a bad impression of myself on people I meet for the first time	0.026	0.384	−0.023	**0.745**	0.170
i21	I act with concern for what others evaluate me	**0.447**	−0.051	0.266	**0.602**	0.114
i16	I am usually aware of my appearance	0.294	−0.042	0.092	0.136	**0.709**
i3	I tend to dress up when others see me	0.181	0.202	−0.110	0.287	0.253
**Private SCS items**						
i15	I often try to make sense of my own mind	−0.021	**0.765**	0.330	0.067	−0.032
i4	I want to grasp the change of my own feelings at the time	0.066	**0.668**	0.088	0.030	−0.061
i1	I am always trying to figure myself out	0.027	**0.660**	−0.006	0.065	0.049
i7	I think about what I really want to do and act on it	0.021	**0.623**	0.046	0.063	−0.137
i17	I always try to remember to look at myself	0.058	**0.586**	0.390	0.110	0.152
i5	I do not really care about what is going on inside me	−0.297	**−0.586**	−0.177	0.010	−0.118
i11	I often take a moment to look at myself from a distance	−0.178	0.267	**0.780**	−0.180	0.119
i14	I often look at myself as I look at others	0.123	0.226	**0.574**	0.021	−0.005
i12	I reflect about myself a lot	0.088	0.085	**0.445**	−0.011	0.051
i20	I am sensitive to changes in my mood	−0.049	−0.045	0.213	0.109	0.252

Note: Factor 1: concern about the self observed by others; Factor 2: internal state awareness; Factor 3: self-reflectiveness from a third-person perspective; Factor 4: concern about being evaluated by others; Factor 5: concern about one’s own appearance. Factor loadings with absolute values higher than 0.4 are shown in bold.

**Table 3:** Results of ROI analysis.

ROIs	Number of Voxels	Cluster-Level *p*-Value (Corrected)	MNI Coordinates			T-Value
			x	y	z	
Brain regions with gray-matter expansions in individuals with high scores on Factor4						
ACC/MCC	1342	0.01	11	20	26	3.74
						
Insula	534	0.07	41	−14	3	3.55
						
Precuneus/cuneus	1080	0.03	12	−48	20	4.12
						
MPFC	633	0.08	−5	42	−21	3.82

Abbreviations: ROI, region of interest; ACC, anterior cingulate cortex; MCC, middle cingulate cortex; MPFC, medial prefrontal cortex; MNI, Montreal Neurological Institute.

## Data Availability

The data that support the findings of this study are available on request from the corresponding author. The data are not publicly available due to their containing information that could compromise the privacy of research participants.
